# Supplementary material for: Evaluation of Systemic Antifungal Prescribing Knowledge and Practice in the Critical Care Setting among ICU Physicians and Clinical Pharmacists: A Cross-Sectional Study
Source: Antibiotics (Basel). 2023 Jan 23;12(2):238. doi: 10.3390/antibiotics12020238 (PMC9952359; doi:10.3390/antibiotics12020238)
Supplement: Supplementary file 1 [file antibiotics-12-00238-s001.zip › antibiotics-2119084-supplementary.pdf]

Supplementary material:

Table S1: Assessment of antifungal agents' pharmacokinetics knowledge between physicians and pharmacists (N=63).

Knowledge and practice statement	Overall	Physician	Pharmacist	P-value §
**Section B: Antifungal agents pharmacokinetics**	**Correct Answer N (%)**	**Correct Answer N (%)**	**Correct Answer N (%)**	
12. For an ICU patient suffering from urosepsis, and a positive Candida albicans urine culture, which antifungal do you think has a significant urine concentration to start therapy?	49 (77.8%)	34 (81.0%)	15 (71.4%)	0.522
13. Would you replace IV fluconazole with oral fluconazole capsules in ICU patients?	44 (69.9%)	32 (76.2%)	12 (57.1%)	0.151
14. For an ICU patient with a high risk of invasive fungal infection and his Candida score is more than 3, he is a CKD patient and Creatinine Clearance is 30 mL/minute and you decided to give him IV Fluconazole as prophylactic antifungal therapy, what do you think about the dose of IV Fluconazole in this patient according to your practice in a hospital?	42 (66.7%)	28 (66.7%)	14 (66.7%)	1.000
15. If you ever come across a case of invasive candidiasis in a pregnant female, which antifungal agent offers the safest option?	12(19%)	03 (07.1%)	09 (42.9%)	**0.001** **
16. This antifungal agent is known for its inherent renal toxicity	38(60.3%)	24 (57.1%)	14 (66.7%)	0.588
17. This class of antifungal drugs usually does not require renal dose adjustments or precautions	41 (65.1%)	24 (57.1%)	17 (81.0%)	0.093
18. An azole that needs adjustment in hepatic impairment	13(20.6%)	05 (11.9%)	08 (38.1%)	**0.023** **
19. In the treatment of candidemia by a fluconazole-susceptible Candida albicans, which dose do you usually prescribe?	20(31.7%)	11 (26.2%)	09 (42.9%)	0.252
**Pharmacokinetics of Antifungal therapy score (mean ± SD) ‡**	**4.16±1.59**	**3.88 ± 1.42**	**4.71 ± 1.82**	**0.050 ****

Table S2: Checklist for Reporting Of Survey Studies (CROSS)

**Section/topic**	**Item**	**Item description**	**Reported on page #**
**Title and abstract**			
Title and abstract	1a	State the word “survey” along with a commonly used term in title or abstract to introduce the study’s design.	1
	1b	Provide an informative summary in the abstract, covering background, objectives, methods, findings/results, interpretation/discussion, and conclusions.	1
**Introduction**			
Background	2	Provide a background about the rationale of study, what has been previously done, and why this survey is needed.	1-2
Purpose/aim	3	Identify specific purposes, aims, goals, or objectives of the study.	2-3
**Methods**			
Study design	4	Specify the study design in the methods section with a commonly used term (e.g., cross-sectional or longitudinal).	13
Data collection methods	5a	Describe the questionnaire (e.g., number of sections, number of questions, number and names of instruments used).	14
	5b	Describe all questionnaire instruments that were used in the survey to measure particular concepts. Report target population, reported validity and reliability information, scoring/classification procedure, and reference links (if any).	14
	5c	Provide information on pretesting of the questionnaire, if performed (in the article or in an online supplement). Report the method of pretesting, number of times questionnaire was pre-tested, number and demographics of participants used for pretesting, and the level of similarity of demographics between pre-testing participants and sample population.	14
	5d	Questionnaire if possible, should be fully provided (in the article, or as appendices or as an online supplement).	4
Sample characteristics	6a	Describe the study population (i.e., background, locations, eligibility criteria for participant inclusion in survey, exclusion criteria).	13

	6b	Describe the sampling techniques used (e.g., single stage or multistage sampling, simple random sampling, stratified sampling, cluster sampling, convenience sampling). Specify the locations of sample participants whenever clustered sampling was applied.	13
	6c	Provide information on sample size, along with details of sample size calculation.	14
	6d	Describe how representative the sample is of the study population (or target population if possible), particularly for population-based surveys.	14
Survey administration	7a	Provide information on modes of questionnaire administration, including the type and number of contacts, the location where the survey was conducted (e.g., outpatient room or by use of online tools, such as SurveyMonkey).	13-14
	7b	Provide information of survey's time frame, such as periods of recruitment, exposure, and follow-up days.	13
	7c	Provide information on the entry process:<br>–>For non-web-based surveys, provide approaches to minimize human error in data entry.<br>–>For web-based surveys, provide approaches to prevent "multiple participation" of participants.	13
Study preparation	8	Describe any preparation process before conducting the survey (e.g., interviewers' training process, advertising the survey).	13
Ethical considerations	9a	Provide information on ethical approval for the survey if obtained, including informed consent, institutional review board [IRB] approval, Helsinki declaration, and good clinical practice [GCP] declaration (as appropriate).	14-15
	9b	Provide information about survey anonymity and confidentiality and describe what mechanisms were used to protect unauthorized access.	14
Statistical analysis	10a	Describe statistical methods and analytical approach. Report the statistical software that was used for data analysis.	14

Results

Descriptive results	12	Provide characteristics of study participants, as well as information on potential confounders and assessed outcomes.	13
**Discussion**			
Limitations	14	Discuss the limitations of the study, considering sources of potential biases and imprecisions, such as non-representativeness of sample, study design, important uncontrolled confounders.	13
Interpretations	15	Give a cautious overall interpretation of results, based on potential biases and imprecisions and suggest areas for future research.	14
Generalizability	16	Discuss the external validity of the results.	14
**Other sections**			
Role of funding source	17	State whether any funding organization has had any roles in the survey's design, implementation, and analysis.	14
Conflict of interest	18	Declare any potential conflict of interest.	14
Acknowledgements	19	Provide names of organizations/persons that are acknowledged along with their contribution to the research.	14
